# Artificial Intelligence and Telehealth may Provide Early Warning of Epidemics

**DOI:** 10.3389/frai.2021.556848

**Published:** 2021-01-29

**Authors:** Janan Arslan, Kurt K. Benke

**Affiliations:** ^1^ Centre for Eye Research Australia, University of Melbourne, Royal Victorian Eye and Ear Hospital, East Melbourne, VIC, Australia; ^2^ Department of Surgery, Ophthalmology, University of Melbourne, Melbourne, VIC, Australia; ^3^ School of Engineering, University of Melbourne, Parkville, VIC, Australia

**Keywords:** artificial intelligence, COVID-19, telehealth, virus, digital disease detection, epidemiology, pattern recognition

## Abstract

The COVID-19 pandemic produced a very sudden and serious impact on public health around the world, greatly adding to the burden of overloaded professionals and national medical systems. Recent medical research has demonstrated the value of using online systems to predict emerging spatial distributions of transmittable diseases. Concerned internet users often resort to online sources in an effort to explain their medical symptoms. This raises the prospect that incidence of COVID-19 may be tracked online by search queries and social media posts analyzed by advanced methods in data science, such as Artificial Intelligence. Online queries can provide early warning of an impending epidemic, which is valuable information needed to support planning timely interventions. Identification of the location of clusters geographically helps to support containment measures by providing information for decision-making and modeling.

## Introduction

The current pandemic involving the COVID-19 virus appears to have overloaded medical systems worldwide, which are nearly at the breaking point due to insufficient resources in the face of the exponential growth of the virus. Thousands of retired medical staff are returning to volunteer their services, but the effort remains concentrated in the urban areas while regional areas are suffering even more from an acute shortage of medical staff. A pre-print from the Peter Doherty Institute for Infection and Immunity recently discussed the rapid production of a national COVID-19 plan using existing influenza pandemic preparedness techniques that have been developed over the course of many years (Moss et al., 2020). Their clinical care modeling study confirmed that the COVID-19 could rapidly overwhelm Australia’s health sector capacity, and re-iterated case targeted measures including isolation of infected individuals and the quarantining of their close contacts. Furthermore, social and physical distancing measures will reduce the number of potential contacts, and in doing so, ease the public health workload (Moss et al., 2020). Preparedness in combination with predictive measures could slow and eventually help to arrest the spread of the virus.

Reviews on internet-based surveillance techniques have been described in the literature (see, for example, Brownstein et al., 2009; Aiello et al., 2020). The techniques used are often based on tracking disease outbreaks by gathering information from the internet, such as from news aggregators, blogs, crowd sourcing platforms and expert-curated discussions. An advantage of using these techniques is that they do not rely on traditional sources of disease surveillance, which are sourced from hospitals and laboratory-based systems from patients who seek care for an illness (Aiello et al., 2020). Rather, these emerging surveillance techniques use real-time data readily available from virtual platforms. Digital disease detection, as it is aptly called, is considered to be an extension of traditional systems but not a replacement (Wilson and Brownstein, 2009; Milinovich et al., 2014). These emerging technologies have thus far proven to be very useful when used alongside traditional methods, but can still be improved to reflect advances in online technologies.

In the last decade, with the rise of machine learning, Artificial Intelligence (AI) applications have grown rapidly and there is increasing interest in analyzing online queries from members of the public, as well as data mining of electronic health records for disease detection. There is untapped potential for using online queries, information discovery and predictive analytics. There is also potential for the combination of internet software tools with AI and Telehealth in the developing world, including Africa, Middle East and South-East Asia. For example, the COVID-19 crisis has been discussed recently in the context of different geographical regions with respect to such issues as AI, big data, blockchain and internet of things (IoT) (Ting et al., 2020). Another recent review highlighted studies using AI to support identification of disease outbreaks to support policy development in low income and medium income countries, noting such methods as expert systems, machine learning, and natural language processing (Schwalbe and Wahl, 2020).

## Potential of Artificial Intelligence and Telehealth

To help mitigate the social and economic impacts of the virus, the use of AI has many attractions. Although humans contribute empathy and intuition to medicine and public health practice, they are biological creatures prone to fatigue, stress, depression, and an assortment of physical and emotional concerns that would affect online search performance. These human qualities can lead to errors in judgment. AI can help to automate detection and localization of virus clusters online and free scarce professional staff for other duties. The high throughput of automated systems could provide valuable information very early in an epidemic that could lead to timely interventions.

Recent reports have revealed that AI algorithms may be able to augment or replace some human-based processes. For example, the United States Center for Disease Control and Prevention warned about the virus on January 6th, 2020. Three days later, the World Health Organization (WHO) made a similar announcement to the public. However, a Canadian AI health monitoring platform known as *BlueDot*–an AI algorithm that sifts through foreign language news, airline ticketing, animal and plant disease networks, and official proclamations–forewarned of the impending pandemic on December 31st, 2019 (Niiler, 2020).

There have been other AI developments in response to COVID-19. For example, an online AI-based diagnostic tool has been developed by the Sydney start-up DetectED-X. The online tool analyses computed tomography (CT) sectional scans of the human torso and rapidly identifies the presence of COVID-19 in the lungs (Detected-X, 2020). Another example is the portable AI device *FluSense*–which was modified for COVID-19 tracking. This was originally designed to analyze influenza trends by detecting and collecting real-time coughing data, along with crowd sizes, noting that coughing is a primary symptom of infection (University of Massachusetts Amherst, 2020). Thermal-imaging is now being used routinely to identify individuals with a fever at various public screening points (Ting et al., 2020).

In addition to diagnostic imaging tools and portable devices, clues on the spread could be obtained by using a number of online queries relating to the combination of targeted search keywords, such as fever, coughing, and recent international travel (using an expert system based on Boolean logic). A regional map of the number of queries could provide information discovery, such as the location of local clusters. There are several influenza-based applications which could be used as a launching pad for COVID-19 AI developments. For example, Deiner and co-workers detected the spatial spread of a conjunctivitis epidemic by tracking online queries on disease symptoms by users of social media such as Google Trends and Twitter (Deiner et al., 2016). A simple example of AI was used in the form of Boolean logic to process the queries. The survey was highly specific to tweets associated with eye disease relating to conjunctivitis symptoms and involved filtering of word content. A further illustration of using internet-based trends and AI-based modeling can be found in Teng et al. (Teng et al., 2017). The authors used Google Trends for the surveillance of the Zika virus. Their study found strong correlations between online searches related to the virus and actual number of reported cases using a machine learning and autoregressive integrated moving average (ARIMA) model.

In the preceding examples, we can identify online platforms, such as Google Trends, as being useful data mining tools for providing data analytics. There are different schools of thought with respect to the application of virtual data for disease detection. On one hand, *BlueDot’s* CEO believes social media data is “too messy” to be used in digital disease detection (Niiler, 2020). However, social media networks are providing first-hand information, and are valuable data sources, even if the data are not being collected with a health objective (Denecke, 2017). Thus, researchers, are using social media posts (together with news reports and data from official public health channels) along with machine learning and natural language processing to parse through available data sources and detect mentions of specific COVID-19-related symptoms (Knight, 2020).

**FIGURE 1:**
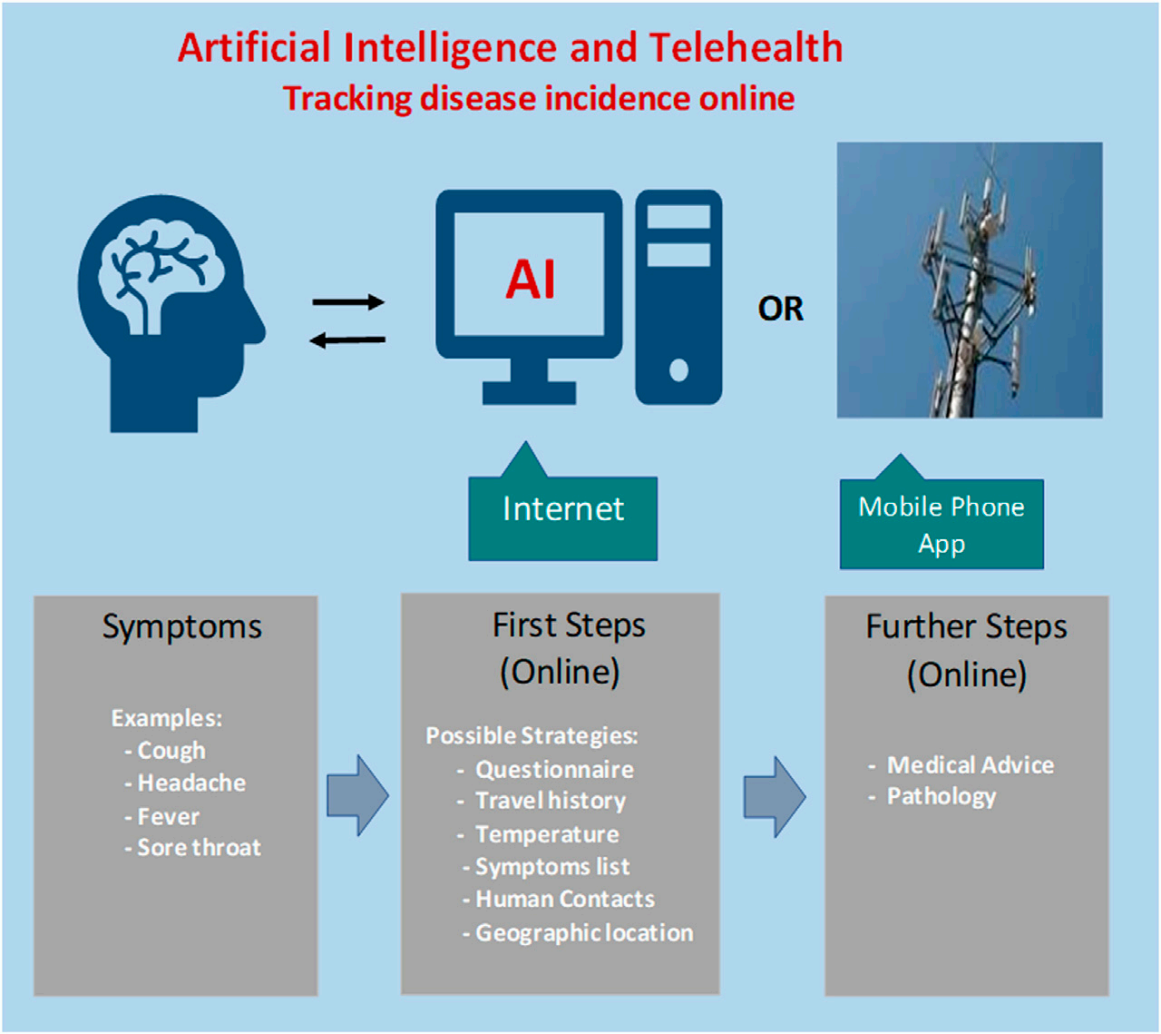
Steps for designing an artificial intelligence-based tracking procedure for regional incidence of COVID-19 virus activity. Concerned citizens with possible symptoms could login and answer a series of questions. Feedback would indicate whether further follow-up is warranted.

The advantage of collating statistics for online queries and social media postings include early warning and the localization of biosecurity threats and epidemics to enable timely intervention by medical authorities. A conceptual process to serve as a baseline for development of an AI-based approach for early warning of an emerging epidemic is depicted in Figure 1.

Following initial screening by online AI, suspicious cases could be referred to medical staff for further investigation. The AI algorithm has the computational power to meet and even exceed the performance of a human doctor in diagnostics, but is missing intuition and soft skills, which are desirable in clinical management, patient guidance and support.

**FIGURE 2:**
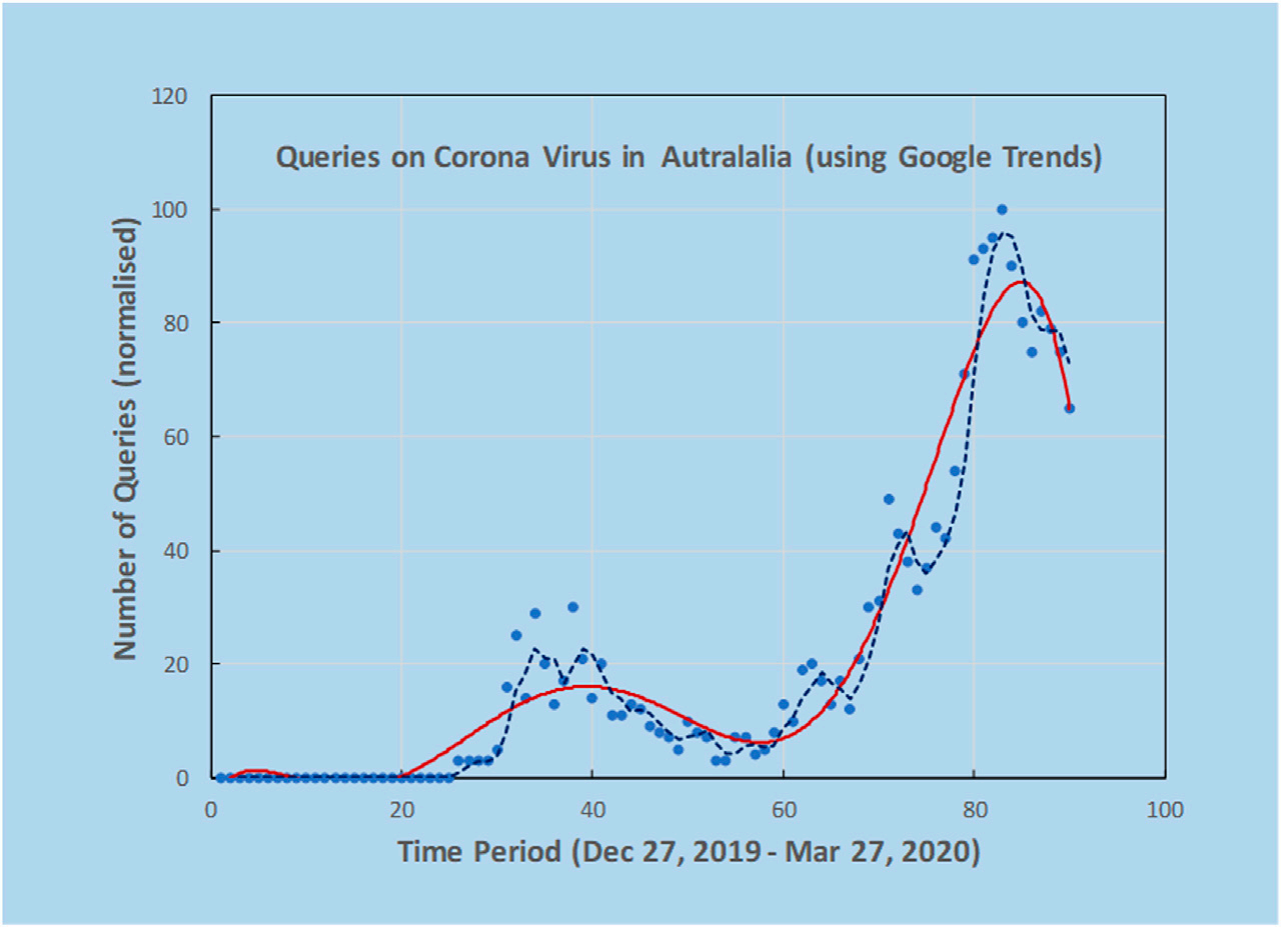
Using Google Trends, it was possible to investigate online search activity over a period of three months using the keywords “Corona Virus Australia”. Solid line is an estimate of trend by fitting a polynomial curve to the scatterplot data from Google Trends and the dashed line is 3-point moving average. Peaks indicate high interest at certain dates that could be correlated with news reports. Note the double peak in query dates.

**FIGURE 3:**
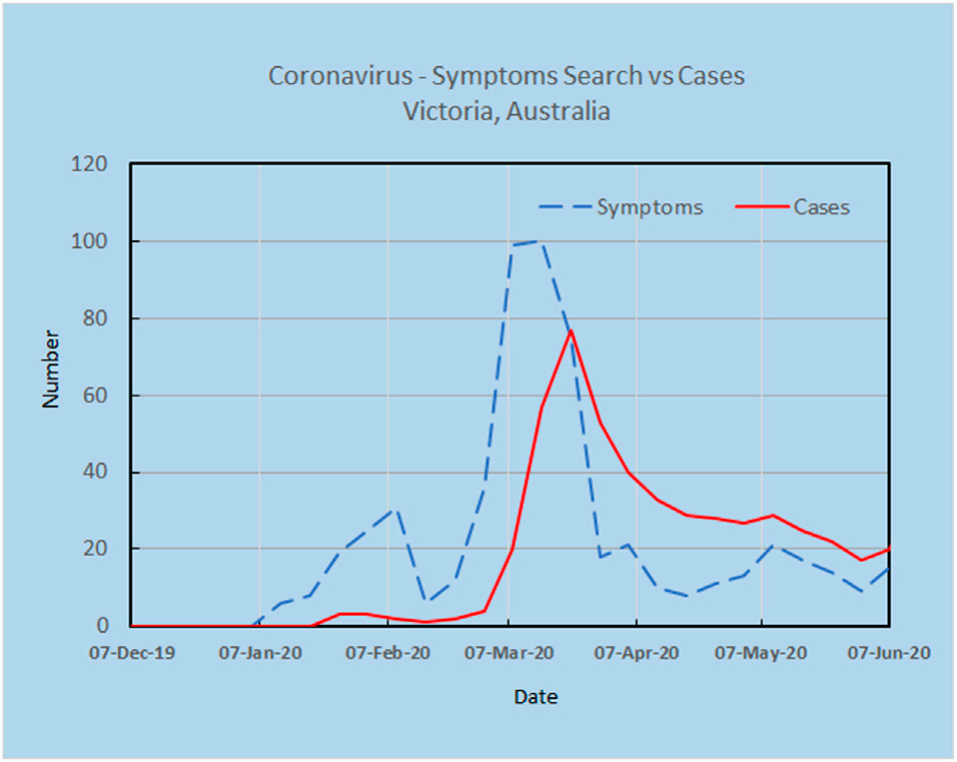
Online search for symptoms reached a maximum during 8–15 March, while the peak for cases was 22 March, providing 1–2 weeks warning.

As an example, a preliminary analysis carried out by the authors using Google Trends and the search terms “Corona Virus Australia” reveals an increasing trajectory of COVID-19 concerns in Australia from late December 2019 to March 2020 (Figure 2). Using AI techniques alongside these preliminary trends, the potential spread and direction of COVID-19 may be estimated before actual confirmed cases begin to surface. This is shown in Figure 3, where the search for symptoms over a six-month period reached a maximum during 8–15 March, while the peak of cases occurred at 22 March, thus providing 1–2 weeks prior warning. The search terms employed were subject to analysis by Boolean logic and were “Victoria AND coronavirus AND symptoms” (Source: Google Trends, Accessed 4 July 20). The results are indicative that monitoring online queries on symptoms from the public may reveal new outbreaks, with prior warnings of 1–2 weeks, which is significant given the exponential growth rate of the virus. These results are from a pilot study only and need to be confirmed with larger trials. There is a need to study the design and structure of search queries to remove ambiguities and to ascertain whether the search performance changes as the epidemic evolves.

Apart from Google Trends, there are also other social media platforms, such as Twitter, that could be used to track COVID-19 or other epidemics. For example, Paul and co-workers describe influenza forecasting using data collected from the Twitter community (Paul et al., 2014). The authors compared this form of forecasting to the more traditional and gold-standard of reporting (i.e., historical influenza-like illness (ILI) data from the CDC). The ILI data has the disadvantage that there is a lag of 1–2 weeks (i.e., time between a patient being diagnosed to when their data appears in the ILI report). The initial reports are often riddled with inaccuracies which are corrected by the CDC over time. Using a basic linear autoregressive model, the authors found that a model incorporating Twitter data outperformed an equivalent model relying solely on ILI data. They found that incorporating Twitter data can reduce influenza forecasting error by 17–30% over a baseline that uses only historical data. Additionally, Twitter can forecast 2–4 weeks ahead of models incorporating historical data. Such findings were mirrored in several other publications, of which we present two for brevity. Achrekar et al. (Achrekar et al., 2011), who found a correlation between the number of flu-related Tweets and reported ILI cases using a autoregressive model with exogenous inputs. Also, Signorini et al. (Signorini et al., 2011) used support-vector regression to make quantitative estimates of ILI values using Twitter feeds, and demonstrated that influenza data gathered from Twitter could accurately track reported disease levels.

Despite the promise shown for mining information from postings on social media, there are also uncertainties in data quality worth consideration, which may include accuracy of data extraction, updating periods of search engines, data authenticity, user motivation, and extent of media coverage. There may also be subconscious bias in search terminology by users due to age, gender and ethnicity, together with under-representation by the elderly with limited access to the internet (Benke, 2017). Failure to adequately address some of these issues may have contributed to the spectacular failure of the Google Flu Trends algorithm to detect the non-seasonal A/H1N1 pandemic in 2009 (Ginsberg et al., 2009; Cook et al., 2011). The Google Flu Trends model–a linear regression model with 45 unique search queries - was updated, but later resulted in overestimation for the 2012–2013 influenza season. As a result, Google removed the Google Flu Trends application from the public domain and it is now accessible only to researchers (Aiello et al., 2020).

To improve confidence of online searches, replication of online Google Trends searches together with spatial stratification of sampling may help to improve results. Results may also be improved by considering different AI approaches, or their combinations, including machine learning, neuro-linguistic programming, and sentiment analysis. AI can also use a *continual learning* approach–where the model continually and autonomously learns from a stream of data (Zenke et al., 2017). For example, machine learning may be used to train on the time-series data documenting the number and location of search queries as a means to estimate weights for a predictive model.

## Mobile Phones

Within the context of COVID-19, a team at the University of Oxford (Bourne, 2015) suggested epidemic control through mobile phone tracing (Ferretti et al., 2020). Similar systems have been deployed in Asia, in which a phone app allows a central database to collect data on user movement and coronavirus diagnosis. According to the authors, “By keeping a temporary record of proximity events between individuals, it can immediately alert recent close contacts of diagnosed cases and prompt them to self-isolate.” The Australian Government (2020) has also released an official Coronavirus App. The app allows users to check their symptoms via a questionnaire (e.g., checking whether patients experience breathlessness or drowsiness), and which contains many more features, such as current status of COVID-19 cases in Australia and registering (Australian Government–Department of Health, 2020).

There are now many countries around the world, including those with very high infection rates, using various methods such as mobile phone apps to track locations of mobile phone users. By providing aggregated data, trends of interests are captured. It would be interesting to conduct further research on how the number of queries on symptoms may change during the actual course of an epidemic.

## Conclusion

There are very real and effective benefits arising from using AI and big data, together with online resources, such as Google and Twitter. The contribution of AI is that it can detect patterns and possible clusters from analyzing online queries of symptoms, which is much faster than physical testing of citizens. The ability to provide early warning of a new epidemic even by a few days is critical when there is exponential growth involved and this may lead to faster interventions that may save lives. Although online platforms cannot replace physical testing, they can provide timely information to support modeling the spread and potential trajectory of epidemics.

## Data Availability Statement

The raw data supporting the conclusions of this article will be made available by the authors, without undue reservation.

## Author Contributions

JA and KKB developed the idea and wrote the manuscript. KKB designed the figures.

## Funding

JA is supported by the RB McComas Research Scholarship in Ophthalmology from the University of Melbourne.
